# Circular RNA UBR1 promotes the proliferation, migration, and invasion but represses apoptosis of lung cancer cells via modulating microRNA-545-5p/SSFA2 axis

**DOI:** 10.1080/21655979.2021.2004977

**Published:** 2021-12-07

**Authors:** Peng Su, Feng Mao, Jian Zhang, Hui Zhang, MingBo Wang, YanZhao Xu, ZiQiang Tian

**Affiliations:** a Department of Thoracic Fifth, Fourth Hospital of Hebei Medical University, ShiJiaZhuang City, HeBei Province, China; b Department of Oncology, Shanghai Chest Hospital,Shanghai Jiao Tong University, ShangHai City, 200030, China; c Department of Radiotherapy, The Fourth Hospital of Hebei Medical University (East), ShiJiaZhuang City, HeBei Province, China

**Keywords:** Lung cancer, circular RNA UBR1, microRNA-545-5p, SSFA2, proliferation, migration, invasion, apoptosis

## Abstract

Lung cancer (LC) is a malignant tumor with the highest incidence in the world, and its specific pathogenesis is still unclear. Circular RNAs (circRNAs) are a group of non-coding RNAs that play a key role in the development and progression of various cancers. The expression pattern and function of circRNAs in LC are still not completely distinct. In this study, it was aimed to study the expression and potential mechanism of circ-UBR1 in LC cells. Then it was found that circ-UBR1 was up-regulated in LC cells, and had microRNA (miR)-545-5p binding sites. Meanwhile, it was confirmed by dual-luciferase reporter assay that circ-UBR1 directly bound to miR-545-5p and then repressed its expression. MiR-545-5p was down-regulated in LC cells and refrained its expression by binding to the downstream target gene SSFA2. Knockdown circ-UBR1 or enhancive miR-545-5p repressed A549 cell proliferation, migration, and invasion, but accelerated apoptosis. After transfection with circ-UBR1 low expression vector, upregulation of SSFA2 apparently reversed the depression of reduced circ-UBR1 on cell proliferation, migration, and invasion, and the promotion of cell apoptosis. Further tumor xenograft experiments in nude mice also confirmed that knockdown of circ-UBR1 could increase the expression of miR-545-5p, but decrease the expression of SSFA2, thus alleviating the progression of LC *in vivo*. Therefore, these results fully indicate that circ-UBR1 promotes LC cell proliferation, migration, and invasion, but represses apoptosis via the circ-UBR1 axis, which may be a closely related marker and therapeutic target of LC.

## 1. Introduction

Lung cancer (LC) is an extremely familiar and fatal malignant tumor in lots of countries, with surprising clinical mortality rate [1–3]. Early examination of LC is available to be achieved via analyzing respiratory tissue samples or applying biomarkers in peripheral blood, which consists of blood and urine [4,5]. For example, tumor markers include carcinoembryonic antigen (CEA), neuron-specific enolase (NSE) and cytokeratin 19 fragment antigen 21–1 (CYFRA21-1) [6]. Nevertheless, the detailed research on LC pathogenesis is not perfect, severely restricting the clinical therapy and impactive prevention of LC. Hence, it is pressed for discovering brand-new biomarkers and molecular mechanisms to boost LC therapy.

Circular RNAs (circRNAs), as a peculiar non-coding RNA, are discovered to modulate the advancement of various diseases, covering cancer [7,8]. CircRNAs are available to perform as a sponge of microRNAs (miRNAs) and indirectly modulate downstream genes, thus mediating the metastasis and other biological functions of cancer cells [9,10]. For instance, circ_0001073 is reported to repress LC advancement via miR-626/LIFR axis, clarifying the latent value of circ_0001073 in LC therapy [11]. Meanwhile, a study proposes that circ-CCS is elevated in LC and forecasts unpleasing prognosis [12]. Currently, molecular targeted therapy clarifies enormous potential in tumor therapy [13,14]. Hence, elucidating the character and mechanism of circRNAs in tumor is promising to offer a novel approach for LC therapy. A former study manifests enhancing circ-UBR1 in breast cancer, and silencing one is available to repress advancement of breast cancer cell with curbed metastasis [15]. Nevertheless, the mechanism of circ-UBR1 in LC advancement has not been explored.

MiRNAs are available to negatively modulate target genes via combining with the 3 ‘untranslated regions of mRNA consisting of complementary sequences [16]. New evidences affirm that miRNAs are linked with a great many cytological processes, like LC growth and apoptosis [17–19]. A former literature manifests the anti-tumor impacts of miR-545-5p on colon cancer [20]. Nevertheless, few studies exist on the mechanism of miR-545-5p in LC occurrence and advancement.

Gene is the carrier of genetic information, and the expressed protein is also the executor of function. SSFA2 is an elevated gene in glioma tissues with expediting glioma advancement [21]. Nevertheless, few studies are present on the mechanism of SSFA2 in LC occurrence and advancement.

In this study, it was proposed for the first time that circ-UBR1 promoted the proliferation, migration, and invasion of LC cells, but repressed apoptosis via modulating miR-545-5p/SSFA2 axis. It was first evaluated the expression of circ-UBR1, miR-545-5p and SSFA2 in LC tissues and cells. Then, their effects on proliferation, migration, invasion, and apoptosis of LC cells were explored. The results clarified that circ-UBR1 and SSFA2 were elevated in LC, while miR-545-5p was reduced. Down-regulation of circ-UBR1 or up-regulation of miR-545-5p repressed proliferation, migration, and invasion of A549 cells, but motivated cell apoptosis. Circ-UBR1 refrained the expression of miR-545-5p, and miR-545-5p negatively modulated SSFA2. Elevated SSFA2 reversed the effects of repressive circ-UBR1 on the biological function of A549 cells. These results suggest that circ-UBR1 may be a potential therapeutic target for LC.

## 2. Materials and methods

### 2.1. Materials

Roswell Park Memorial Institute (RPMI)-1640 medium and Dulbecco’s Modified Eagle Medium (DMEM) were purchased from Gibco (Carlsbad, CA, USA). The sh-NC, sh-UBR1, oe-NC, oe-UBR1, mimic NC, miR-545-5p mimic, inhibitor NC, miR-545-5p inhibitor, pcDNA-NC and pcDNA-SSFA2 plasmid vectors were synthesized via GenePharma Co., Ltd. (Shanghai, China). The cDNA synthesis kit were purchased from Applied Biosystems (Foster City, CA, USA), with primary antibodies from Abcam (Cambridge, MA, USA), TRIzol from Life Technologies (New York, USA), and BCA protein detection kit from Beyotime (Shanghai, China).

### 2.2. Clinical sample collection

Collection of 40 cases of LC and para-cancer tissues was from patients undergoing surgery in The Fourth Hospital of Hebei Medical University. Inclusion criteria included: 1) All clinicopathological diagnoses were confirmed by two pathologists; 2) None of the patients received any treatment before surgery; 3) Patients did not receive radiotherapy or chemotherapy during follow-up; 4) without a history of other concurrent malignancies. Exclusion criteria consisted of: 1) Patients receiving preoperative treatment, including chemotherapy or radiotherapy; 2) Patients with tumor types in other organs. Store of all specimens was at −80°C for further study. Approval of this study was via the Ethics Committee of The Fourth Hospital of Hebei Medical University, and gain of the written informed consent of the above patients was before the study.

### 2.3. Cell culture

Culture of LC cell lines (H1299, H460, H520, A549) and human normal lung epithelial cells (BEAS-2B) (Cell resource center, SIBS, CAS, Shanghai, China) was in Roswell Park Memorial Institute-1640 (Gibco, Carlsbad, CA, USA) (the former three), and Dulbecco’s Modified Eagle Medium (Gibco) (the rest) in a humidified incubator. Supplement of all medium was with 10% Fetal bovine serum (FBS) and 1% penicillin/streptomycin. All the cells were cultured in a humidified incubator containing 5% CO_2_ at 37°C [22].

### 2.4. Cell transfection

GenePharma Co. Ltd. (Shanghai, China) was commissioned for construction of sh-negative control (NC), sh-UBR1, overexpressed (oe)-NC, oe-UBR1, mimic NC, miR-545-5p mimic, inhibitor NC, miR-545-5p inhibitor, pcDNA-NC and pcDNA-SSFA2 plasmid vectors. A549 cells were transfected via applying Lipofectamine 3000 (Life Technologies Corporation, Carlsbad, CA, USA) following the manufacturer’s instructions. Forty-eight hours later, the cells were harvested for subsequent assay.

### 2.5. Quantitative real-time polymerase chain reaction (qRT-PCR)

Using Trizol reagent (Life Technologies, New York, USA), total RNA was isolated from tissues or cells according to standard instructions. Then synthesis of the first strand of complementary DNA (cDNA) was via either TaqMan Reverse Transcription Kit (for circ-UBR1 and SSFA2) or TaqMan miR Reverse Transcription Kit (for miR-545-5p) (Applied Biosystems, Foster City, CA, USA). SYBR Select Master Mix (Roche, Basel, Switzerland) was applied to perform qRT-PCR on CFX Connect Real-Time PCR System (Bio-Rad, Hercules, CA, USA). The reaction conditions were 95°C, 2 min; 95°C, 30 s, 60°C, 30 s and 72°C, 30 s, and 40 cycles [23]. Normalization of relative expression was to glyceraldehyde-3-phosphate dehydrogenase (GAPDH) or U6, and calculation of the expression was via 2^−ΔΔCt^. Each experiment was repeated three times. Primer sequences are clarified in Table 1.

**Table 1.** Primers for RT-qPCR

Genes	Primer sequences (5ʹ – 3ʹ) PCR product
Circ-UBR1	F: TCTGTGCAATACTGTGATCCCC 132bp
	R: GGAGCTTTTTGAAGCTGTTGCT
MiR-545-5p	F: AGCGCGTCAGTAAATGTTTATT 110bp
	R: GTTGTTGGTTGGTTGGTTGT
SSFA2	F: TGGCAAGAAAGGCCCCTGTG 100bp
	R: GGAGCAGCAGCAGGATCAGG
U6	F: CTCGCTTCGGCAGCACA 108bp
	R: AACGCTTCACGAATTTGCGT
GAPDH	F: CACCCACTCCTCCACCTTTG 95bp
	R: CCACCACCCTGTTGCTGTAG

### 2.6. Western blot

After harvest of the cells, lysis was with Radio-Immunoprecipitation assay lysis buffer (Beyotime, Shanghai, China) covering phenylmethylsulfonyl fluoride (Beyotime) to gain total protein. Application of bicinchoninic acid protein assay kit (Beyotime) was for determination of the protein concentration. An equal amount (40 μg) of protein was resolved by 8%–10% sodium dodecyl sulfate polyacrylamide gel electrophoresis (SDS-PAGE). Electroblot of the gel was onto polyvinylidene fluoride membranes (0.2 μm, Beyotime). Behind seal in 5% skim milk (Sangon Biotech, Shanghai, China), incubation of the membrane was with primary antibodies: SSFA2 (1: 1000) and GAPDH (1: 1000) (both Abcam, Cambridge, MA, USA), and horseradish peroxidase coupled secondary antibody (Abcam). Finally, observation of all protein bands was via an enhanced chemiluminescence system (Millipore, Billerica, MA, USA). Normalization of protein abundance was via GAPDH and assessment of band density was via ImageJ software. Each experiment was repeated three times [24].

### 2.7. Cell counting Kit −8 (CCK-8)

CCK-8 was for detection of cell proliferation. Seeding of the cells was with 5.0 × 10^3^ cells/mL in 96-well plates for 24, 48 and 72 h. At the end of each time point, the cells were stained with 10 μL CCK-8 solution (Dojindo, Tokyo, Japan) and incubated at 37°C for3 h. Measurement of the absorbance was with a spectrophotometer at 450 nm. Each experiment was repeated three times [25].

### 2.8. Colony formation assay

Behind transfection, seeding of A549 cells was into 6-well plates at a density of 1000 cells per group and culture for 14 days was to form colonies. Fixation of the resulting colonies was then with 4% formaldehyde (P804536, Macklin, Shanghai, China) and then stain was with 0.3% crystal violet. After removing the excess crystal violet, count of visible colonies and analysis were via Image J software 1.8.0. Each experiment was repeated three times [18].

### 2.9. Flow cytometry

The cells from each group were collected and suspended in 500 μL binding buffer. Then, the cells were stained with 5 μL Annexin V-fluoresceinisothiocyanat (FITC) and 5 μL propidium iodide at room temperature and incubated in the dark for 15 min. Finally, apoptosis was detected by flow cytometry. Meanwhile, there were three replicates of each experiment [26].

### 2.10. Wound healing assay

After transfection, culture of A549 cells was in a 6-well plate with 3.5 × 10^5^ cells in 2 mL complete medium until cell confluence reached 95%. Then, application of 20 μL pipette tips was to form vertical wounds in each well, which was then supplemented with FBS-free medium. Via phase-contrast optical microscope (Axio Lab. A1 Pol; Leica, Solms, Germany), collection of the images was from each well at 0 and 48^th^ h. Application of Image J software 1.8.0 (National Institutes of Health, Bethesda, USA) was for analysis of the images in the determination. Each experiment was repeated three times [27].

### 2.11. Transwell

Matrigel Transwell chamber (BD) full of serum-free medium was for invasion determination. Load of the upper chamber was with 1 × 10^4^ cells. Medium consisting of 20% FBS was employed as a chemical attractant for the lower chamber. After incubation at 37°C for 24 h, fixation of the cells adhered to the lower membrane, stain, count was via a microscope and a counting chamber (Olympus, Tokyo, Japan). Three replicates were presented in each experiment [28].

### 2.12. The luciferase activity assay

In line with the forecasted and mutational-binding sites of circ-UBR1 and SSFA2 3ʹuntranslated region (UTR) in miR-545-5p, construction of wild- (WT) and mutant types (MUT) -circ-UBR1 and SSFA2 3ʹUTR vectors was via pmirGLO reporter vectors (Promega, Madison, WI, USA). Co-transfection of A549 cells was with miR-545-5p mimic or its NC and these reporter vectors. After transfection for 48 h, detection of the luciferase activity of the cells was via dual-luciferase reporter gene assay kit (Promega, Madison, WI, USA). Each experiment was repeated three times [12].

### 2.13. Tumor xenograft in nude mice

To measure the effects of circ-UBR1 *in vivo*, 1 × 10^6^ A549 cells and 20 μL sh-UBR1 were injected into 5-week-old female nude mice (NU/NU Crl: NU-Fox1nu, Charles River Laboratories; Sulzfeld, Germany). In addition, 1106 cells were re-suspended in phosphate buffered saline (PBS; 1: 1 mixed with Matrigel, Corning), and subcutaneously injected into the left flank of mice. Tumor volume was measured every 7 days and the animals were euthanized before the tumor diameter reached 10 mm. Tumor volume V (mm^3^) = (width × length)^2^/2. The research was approved by the Animal Care and Use Committee of our hospital and was conducted in accordance with the guidelines of the National Institute for Animal Care and Ethics. The mice inoculated with the cells were euthanized 5 weeks later. The tumor weight was evaluated and the expression of circ-UBR1, miR-545-5p and SSFA2 was measured [23].

### 2.14. Data statistics

Processing of all data was via SPSS 21.0 statistical software (SPSS, Inc, Chicago, IL, USA). Presentation of the measurement data was mean ± standard deviation (SD). In the measurement data subject to normal distribution, t test was employed for two-group comparison, with one-way analysis of variance (ANOVA) for comparison among multiple groups and Tukey’s post hoc test. Comparison of the data of groups at different time points was via repeated measuring ANOVA and Bonferroni post hoc test. To evaluate the relationship between circ-UBR1 and survival prognosis in patients with LC, Kaplan–Meier method was applied for survival analysis and logarithmic rank test was employed to determine statistically significant differences between high and low expression curves. Pearson correlation analysis was on the association of circ-UBR1 and miR-545-5p, miR-545-5p and SSFA2 in clinical samples. *P* < 0.05 was accepted as indicative of manifest differences [12].

## 3. Results

### 3.1. Elevated circ-UBR1 is in LC; reduced one restrains A549 cell advancement

**Figure 1.**
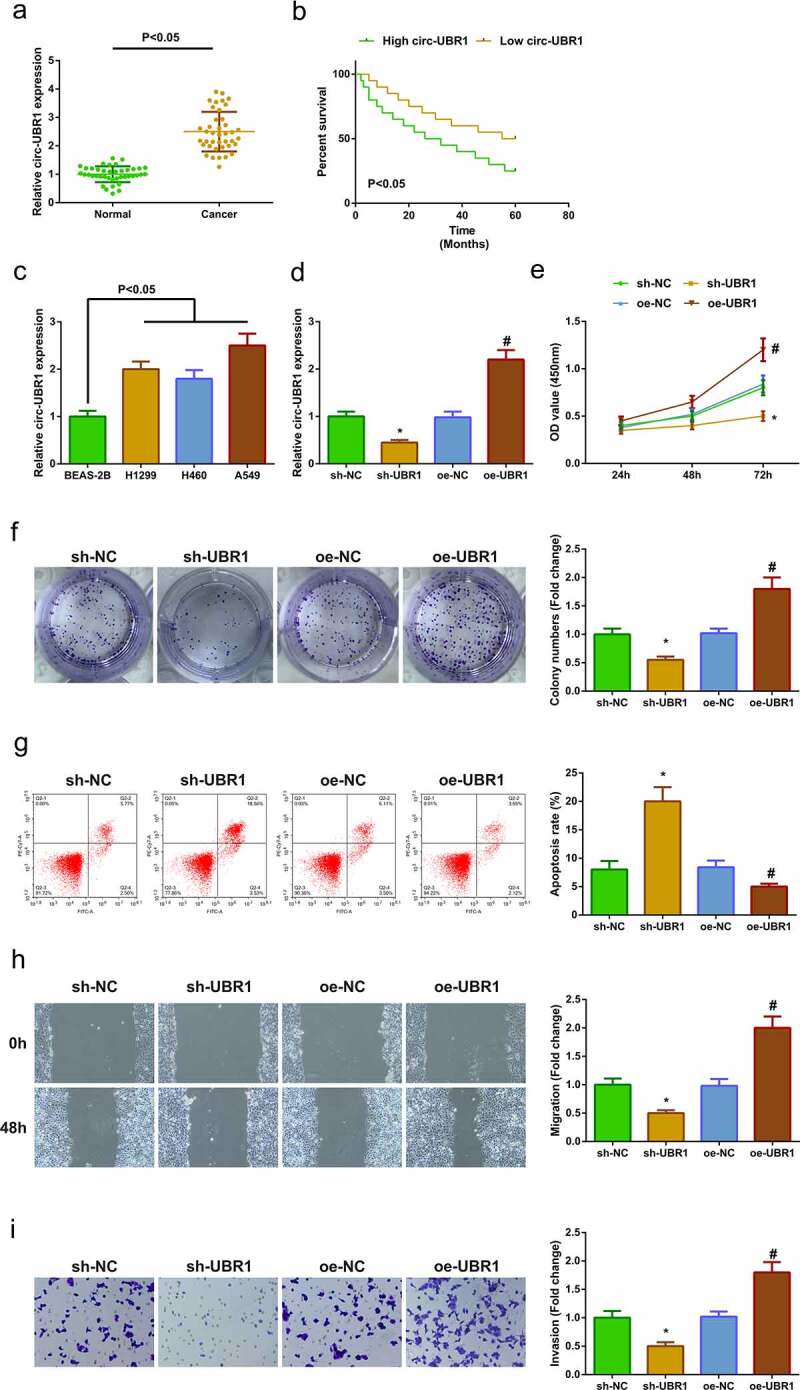
**Elevated circ-UBR1 is in LC, and repressive one refrains A549 cell advancement**. (a) qRT-PCR detection of circ-UBR1 in LC and adjacent tissues (n = 40); (b) Survival prognosis analysis; (c) qRT-PCR to detect the expression of circ-UBR1 in LC cell lines; (d) qRT-PCR to detect circ-UBR1 expression in A549 cells after circ-UBR1 expression intervention; (e/f) After intervention of circ-UBR1 expression, CCK-8 and Colony formation assay to detect cell proliferation; (g) After intervention of circ-UBR1 expression, Cell apoptosis detected via flow cytometry; (h) After intervention of circ-UBR1 expression, Cell migration detected via wound healing assay; (i) After intervention of circ-UBR1 expression, Transwell to detect cell invasion. The data in the Fig. were all measurement data, and manifestation of the values was mean ± SD. * vs the sh-NC, *P* < 0.05; # vs the oe-NC, *P* < 0.05

For exploring circ-UBR1 in LC tissues, detection of circ-UBR1 was conducted, clarifying the elevation (Figure 1(a)). Via analyzing the clinical information table (Table 2), it was discovered that circ-UBR1 was linked with Tumor Node Metastasis staging and lymph node metastasis, and elevated circ-UBR1 forecasted unpleasing prognosis (Figure 1(b)). Meanwhile, circ-UBR1 was also enhancing in LC cell lines, among which the A549 cell line was most apparently repressed (Figure 1(c)). Hence, choice of A549 cells was conducted for subsequent experiments.

**Table 2.** Association of circ-UBR1 with clinical features in LC patients

		Circ-UBR1		
Features	Number	High (n = 20)	Low (n = 20)	*P*
Age				0.752
<60	20	9	11	
60 or more	20	11	9	
Gender				0.748
Male	24	11	13	
Female	16	9	7	
Smoking Status				0.273
Ever	30	13	17	
Never	10	7	3	
Histological classification				0.725
SCC (Squamous cell carcinoma)	15	8	7	1.000
AD (adenocarcinoma or other)	25	12	13	
Tumor Size				0.301
3 cm or less	12	4	8	
>3 cm	28	16	12	
TNM Stage				<0.05
I + II	13	1	12	
III + IV	27	19	8	
Lymphatic metastasis				<0.05
Yes	31	12	19	
No	9	8	1

To further study the character of circ-UBR1 in LC, transfection of sh/oe UBR1 was into A549 cells, with verification of successful transfection (Figure 1(d)). The cell proliferation was detected by CCK-8 and plate cloning, and the experimental results showed that the proliferation ability was apparently decreased after down-regulation of circ-UBR1 (Figure 1(e,f)). The cell apoptosis was detected by flow cytometry, and the experimental results clarified that the apoptosis level was elevated obviously after repressive circ-UBR1 (Figure 1(g)). The cell migration and invasion were examined by plate scratches and Transwell, and it was found that down-regulation of circ-UBR1 clearly reduced the cell migration and invasion abilities (Figure 1(h,i)), while up-regulation of circ-UBR1 promoted the growth of A549 cells.(Figure 1(e-i)). Briefly, depressive circ-UBR1 refrained cell advancement, while elevated one took on the adverse influence.

### 3.2. Circ-UBR1 curbs miR-545-5p

**Figure 2.**
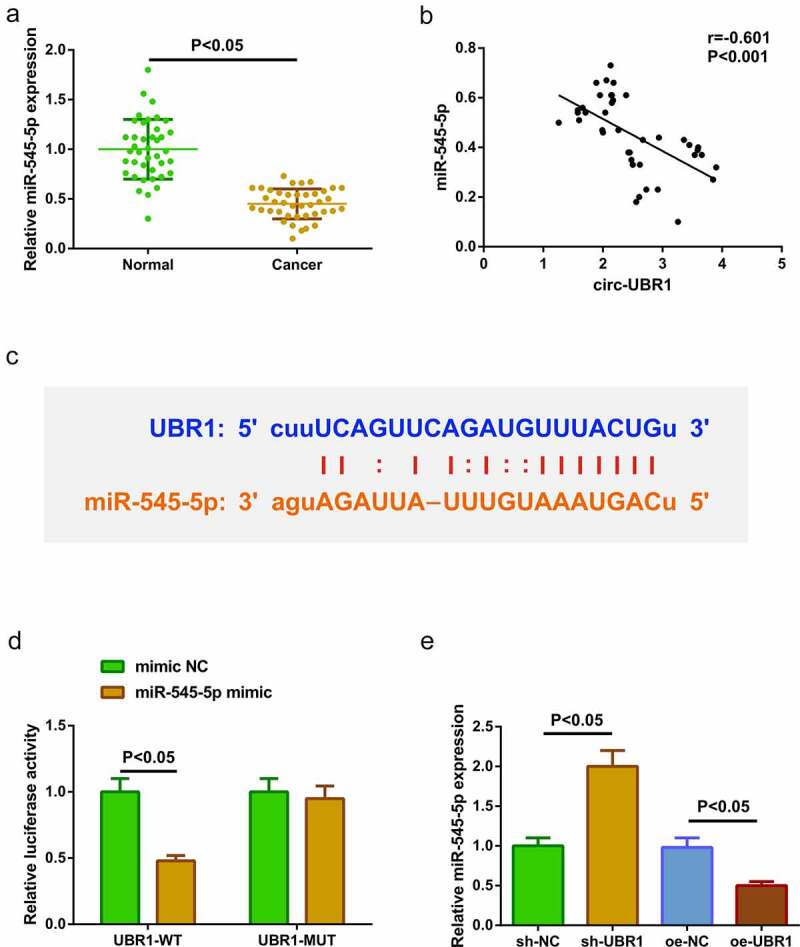
**Circ-UBR1 represses miR-545-5p. (**a) qRT-PCR detection of miR-545-5p in LC and adjacent tissues (n = 40); (b) Association of circ-UBR1 with miR-545-5p; (c) Bioinformatics website starBase to predict the binding site of circ-UBR1 to miR-545-5p; (d) The targeting of circ-UBR1 with miR-545-5p verified via the luciferase activity assay; (e) qRT-PCR detection of miR-545-5p in A549 cells of each group. The data in the Fig. were all measurement data, and manifestation of the values was mean ± SD. *P* < 0.05

For clarifying miR-545-5p in LC tissues, the detection was conducted, manifesting the repression (Figure 2(a)). It was identified elevated circ-UBR1 and reduced miR-545-5p in LC tissues. Hence, it was speculated a binding was in circ-UBR1 with miR-545-5p. Analysis of the association of the factors in clinical samples manifested that circ-UBR1 was negatively linked with miR-545-5p (Figure 2(b)). For further verification, via the bioinformatics website starBase (https://starbase.sysu.edu.cn/agoClipRNA.php) was forecasted the binding site of circ-UBR1 with miR-545-5p (Figure 2(c)). Meanwhile, the luciferase activity was reduced after the co-transfection of UBR1-WT with miR-545-5p mimic (Figure 2(d)). These manifested a binding association was in circ-UBR1 with miR-545-5p. Next, detection of miR-545-5p in A549 cells clarified the repressive one after elevated cicr-UBR1, and the enhancing one behind declined UBR1 (Figure 2(e)). The above experiments testified that circ-UBR1 repressed miR-545-5p.

### 3.3. Elevated miR-545-5p curbs A549 cell advancement, while declined one is adverse

**Figure 3.**
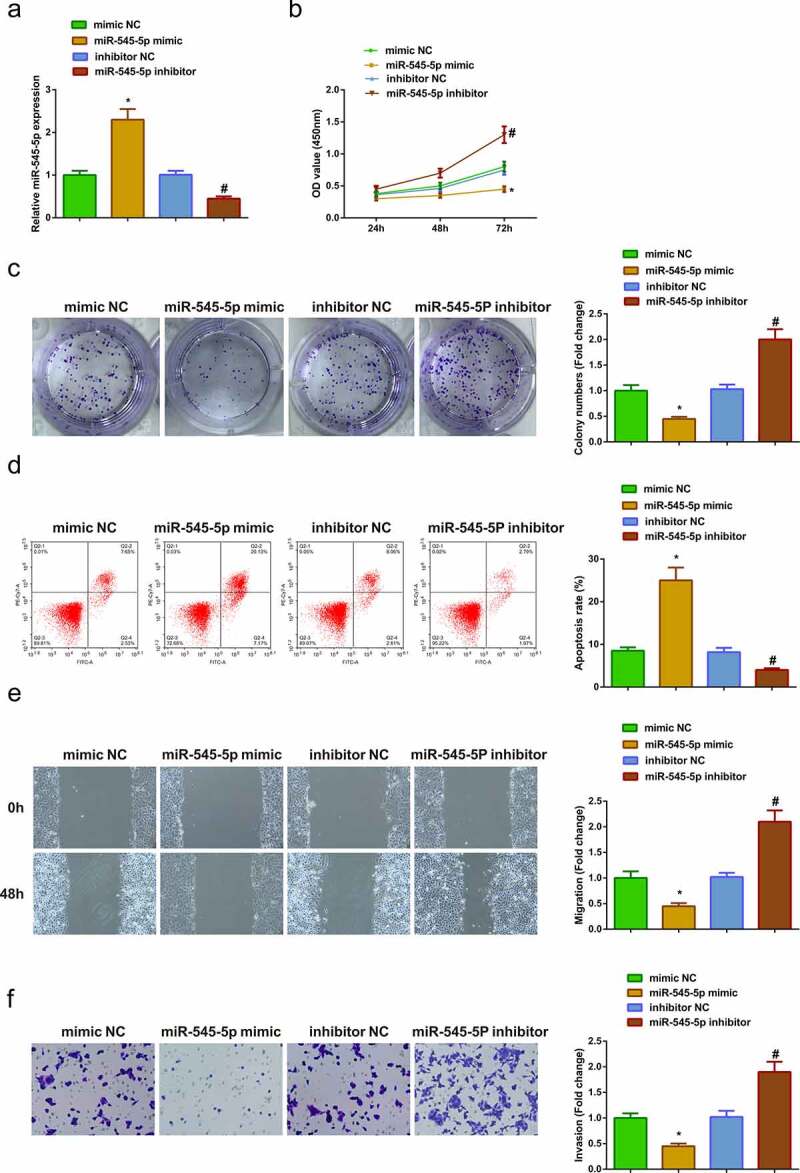
**Up-regulated miR-545-5p curbs A549 advancement, while reduced one is adverse**. (a) After intervention of miR-545-5p expression, qRT-PCR to detect miR-545-5p expression inA549 cells; (b/c) After intervention of miR-545-5p expression, CCK-8 and Colony formation assay to detect cell proliferation; (d) After intervention of miR-545-5p expression, Cell apoptosis detected via flow cytometry; (e) After intervention of miR-545-5p expression, Cell migration detected via wound healing assay; (f) After intervention of miR-545-5p expression, Cell invasion detected via Transwell assay; The data in the Fig. were all measurement data, and manifestation of the values was mean ± SD. * vs the mimic -NC, *P* < 0.05; # vs the inhibitor NC, *P* < 0.05

In order to explore the role of miR-545-5p in LC, A549 cells were transfected with mimic NC, miR-545-5p mimic, inhibitor NC and miR-545-5p inhibitor (Figure 3(a)). Various experiments manifested that upregulation of miR-545-5p could repress the proliferation, migration, and invasion of A549 cells but induce apoptosis, while down-regulation of miR-545-5p could facilitate the growth of A549 cells (Figure 3(b-f)). Shortly, enhancing miR-545-5p curbed A549 cell advancement, while repressive one was opposite.

### 3.4. MiR-545-5p targets SSFA2

**Figure 4.**
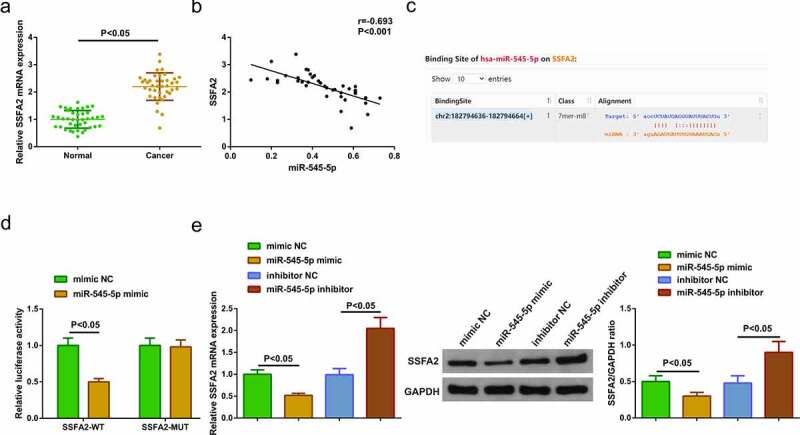
**MiR-545-5p negatively modulates SSFA2. (**a) qRT-PCR and Western blot detection of SSFA2 in LC and adjacent tissues (n = 40); (b) Association of miR-545-5p with SSFA2; (C) Bioinformatics site starBase to predict the target sites of miR-545-5p and SSFA2; (D) The targeting of miR-545-5p with SSFA2 verified via the luciferase activity assay; (e) qRT-PCR and Western blot detection of SSFA2 in A549 cells of each group. The data in the Fig. were all measurement data, and manifestation of the values was mean ± SD. *P* < 0.05

For exploration of SSFA2 in LC tissues, the operation was manifested, clarifying the elevation (Figure 4(a)). It was identified that reduced miR-545-5p and elevated SSFA2 in LC tissues. Hence, it was speculated a targeting was in miR-545-5p with SSFA2. Analysis of the association of the factors manifested that miR-545-5p was negatively linked with SSFA2 (Figure 4(b)). For further verification, via the bioinformatics website starBase (https://starbase.sysu.edu.cn/agoClipRNA.php) was forecasted the targeting binding site of miR-545-5p with SSFA2, with verification of the targeting of the factors (Figure 4(c,d)). Next, detection of SSFA2 in A549 cells clarified the repressive one after elevated miR-545-5p, and the enhancing one behind declined miR-545-5p (Figure 4(e)). The above experiments testified that miR-545-5p negatively modulated SSFA2.

### 3.5. Circ-UBR1/miR-545-5p/SSFA2 axis takes part in LC occurrence and advancement

**Figure 5.**
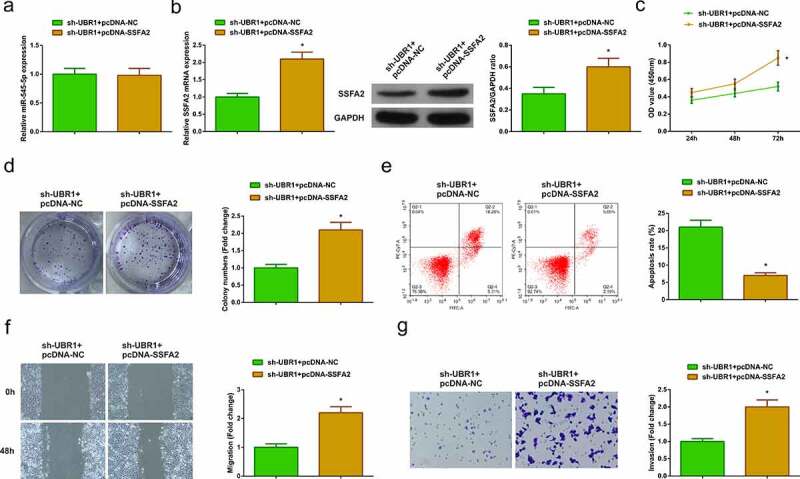
**Circ-UBR1/miR-545-5p/SSFA2 axis participates in LC onset and advancement**. (a/b) qRT-PCR or Western blot to detect miR-545-5p and SSFA2 expression in each group of A549 cells; (c/d) CCK-8/Colony formation assay to detect cell proliferation; (e) Cell apoptosis detected via flow cytometry; (f) Cell migration detected via wound healing assay; (g) Cell migration detected via Transwell assay. The data in the Fig. were all measurement data, and manifestation of the values was mean ± SD. * vs the sh-UBR1 + pcDNA-NC, *P* < 0.05

For further exploration of the modulatory impacts of circ-UBR1/miR-545-5p/SSFA2 axis on LC, transfection of sh-UBR1 + pcDNA-NC/SSFA2 was into A549 cells. The results of qRT-PCR or Western blot manifested that pcDNA-SSFA2 did not reverse the effect of sh-UBR1 on miR-545-5p expression, but clearly reversed the repressive effect of sh-UBR1 on SSFA2 expression (Figure 5(a,b)). In addition, the experimental results manifested that pcDNA-SSFA2 apparently reversed the depressive effect of sh-UBR1 on proliferation, migration, and invasion of A549 cells and the facilitation of apoptosis (Figure 5(c-g)), indicating that circ-UBR1 was involved in the progression of LC by modulating miR-545-5p/SSFA2 axis.

### 3.6. *Circ-UBR1 affects LC tumor growth* in vivo *via regulating miR-545-5p/SSFA2 expression*

**Figure 6.**
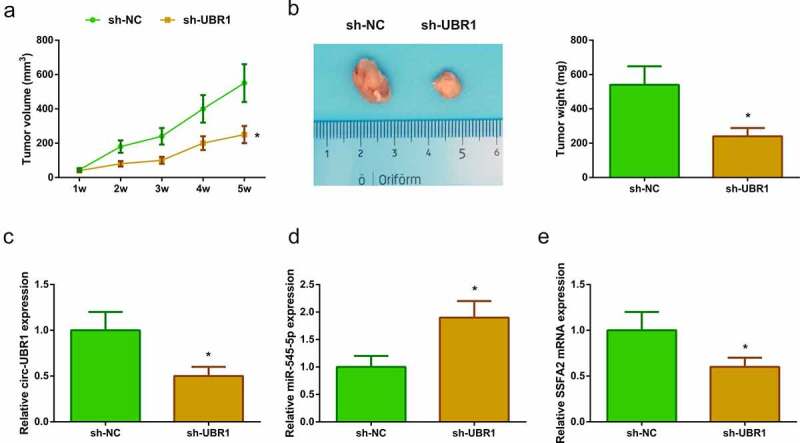
**Circ-UBR1 impacts LC tumor growth *in vivo* by regulating miR-545-5p/SSFA2 expression. (**a/b) Tumor volume and mass of the mice after down-regulating circ-UBR1 (n = 6); (c-e) qRT-PCR or Western blot to detect the expression of circ-UBR1, miR-545-5p or SSFA2 (n = 6); The values were represented by mean ± SD. * vs the sh-NC, *P* < 0.05

To explore the anticancer effect of circ-UBR1 silencing, a xenografted model was established *in vivo* using A549 cells transfected with sh-UBR1 or sh-NC. After 5 weeks of cell injection, tumor volume and weight were reduced in the sh-UBR1 group (Figure 6(a,b)). Meanwhile, circ-UBR1 expression was apparently down-regulated in the sh-UBR1 group (Figure 6(c)). In addition, miR-545-5p expression was elevated in the sh-UBR1 group (Figure 6(d)). However, SSFA2 protein and mRNA expression was reduced in the sh-UBR1 group (Figure 6(e)). These results suggested that circ-UBR1 knockdown might repress LC development via regulating miR-545-5p/SSFA2 expression.

## 4. Discussion

As a prevalent cancer, LC has brought grievous physical, mental and economic burdens to patients and their families, and also become a severe social health problem. A better understanding of the features of LC is crucial to develop brand-new cancer therapies. There are literatures summarizing the classic characteristics of cancer: avoidance of apoptosis, tissue invasion and metastasis, unlimited replication potential and so on [29,30]. The essence of cancer therapy is to combat these features of cancer. Elevated evidences clarify that disparate circRNAs expressed in cancer frequently forecast the diverse functions in cancer. A study has proposed that circ-IGF1R represses LC advancement via the latent circ-IGF1R-miR-1270-VANGL2 network [31]. Another study has clarified that hsa_circ_0062389/miR-103a-3p/CCNE1 axis is beneficial for tumor formation in NSCLC [32]. Meanwhile, circ-ABCB10 knockdown boosts cisplatin sensitivity of LC cells via targeting miR-556-3p/AK4 axis [33]. The study confirmed the high expression of circ-UBR1 in clinical tissues and cells, suggesting that circ-UBR1 plays an important role in LC. To prove this hypothesis, a series of studies were conducted and the results clarified that circ-UBR1 silencing could repress the proliferation, metastasis and invasion, but promote apoptosis of LC cells, while enhancive circ-UBR1 had the opposite effect. This takes on that circ-UBR1 is supposed to be linked with LC advancement as an oncogene.

MiRNAs are abnormally manifested in great many human cancers and play crucial functions, so they have enormous potential as diagnostic biomarkers and therapeutic targets in cancer [34]. MiRNAs have been confirmed to be vital in the physiological and pathological processes of LC and recognized as a latent biomarker for LC diagnosis and prognosis [35]. The study manifested that circ-UBR1 could target miR-545-5p. In the meantime, the expression of miR-545-5p in LC tissues was also reduced than that in normal tissues. The study proved that miR-545-5p might also be involved in the modulation of LC progression. It was confirmed that elevated miR-545-5p apparently repressed LC cell proliferation, migration, and invasion, but accelerated apoptosis, suggesting its antitumor effect in LC.

SSFA2, also named as KRAP, has been discovered to be essential in malignant tumors [36–38]. SSFA2 is supposed to be a cytoskeleton-linked protein referring to structural integrity and/or signal transduction in human cancers [39]. A study has discovered the participation of KRAP in controlling filamentous actin and extracellular signaling [40]. A former study has clarified that loss of SSFA2 results in declined IGF1 protein in mice [41]. Insulin-like growth factor (IGF) is an active protein polypeptide substance necessary for the physiological impacts of growth hormone, consisting of IGF1 and IGF2. As an extracellular ligand, IGF combines with receptors on the cell membrane via all kinds of secretory ways, like IGF-1 R and IGF-2 R to manifest its biological character. Among them, IGF-1 R mediates great many biological reactions of IGF-1 and −2. IGF-1 R is affiliated from the tyrosine protein kinase family and extensively manifested on lots of cell surfaces. Free IGF combines with IGF-1 R, inducing autophosphorylation of receptor β subunit tyrosine, triggering multiple downstream signal activation responses. Present studies consist of the PI3K/AKT and Ras/MAPK pathways, which can induce cell progression [42,43]. The study confirmed the targeting relationship between SSFA2 and miR-545-5p, and testified also that SSFA2 expression in LC tissues was higher than that in normal tissues. Further studies confirmed that up-regulation of SSFA2 reversed the repressive effect of depressive circ-UBR1 on proliferation, migration, and invasion, as well as the promoting effect on apoptosis of LC cells. Further tumor xenograft experiments in nude mice also testified that knockdown circ-UBR1 could elevate the expression of miR-545-5p but decrease the expression of SSFA2, thus alleviating the progression of LC *in vivo*. In short, the results affirm that circ-UBR1 functions in LC via modulating the miR-545-5p/SSFA2 axis.

Nevertheless, still several limitations are in this study. First, the character of circ-UBR1/miR-545-5p/SSFA2 axis *in vivo* was not explored in this study. Stably expressed LC cell lines are constructing to explore the impacts of circ-UBR1/miR-545-5p/SSFA2 axis on tumor growth. Meanwhile, further study of the character of SSFA2’s downstream pathway (PI3K/AKT or Ras/MAPK) in LC was not manifested. This is an interesting direction that will be explored in later studies.

## 5. Conclusion

In summary, the study of miR-545-5p/SSFA2 axis elucidates the carcinogenic role of circ-UBR1 in the progression of LC. This mechanism may be beneficial to the cognition of the pathogenesis of LC. Circ-UBR1 is considered as a useful biological target for early diagnosis and late treatment of LC patients.
